# Comparative effectiveness of natalizumab on cognition in multiple sclerosis: A cohort study

**DOI:** 10.1177/13524585231153992

**Published:** 2023-02-15

**Authors:** Ali Manouchehrinia, Hanna Larsson, Mohammad Ehsanul Karim, Jan Lycke, Tomas Olsson, Ingrid Kockum

**Affiliations:** Department of Clinical Neuroscience, Karolinska Institutet, Stockholm, Sweden/Centre for Molecular Medicine, Karolinska University Hospital, Stockholm, Sweden/The Karolinska Neuroimmunology & Multiple Sclerosis Centre, Department of Clinical Neurosciences, Karolinska Institutet, Centre for Molecular Medicine, Stockholm, Sweden; Department of Clinical Neuroscience, Karolinska Institutet, Stockholm, Sweden/Centre for Molecular Medicine, Karolinska University Hospital, Stockholm, Sweden; School of Population and Public Health, The University of British Columbia, Vancouver, BC, Canada/Centre for Health Evaluation and Outcome Sciences, University of British Columbia, Vancouver, BC, Canada; Department of Clinical Neuroscience, Institute of Neuroscience and Physiology, Sahlgrenska Academy, Gothenburg University, Gothenburg, Sweden/Department of Neurology, Sahlgrenska University Hospital, Gothenburg, Sweden; Department of Clinical Neuroscience, Karolinska Institutet, Stockholm, Sweden/Centre for Molecular Medicine, Karolinska University Hospital, Stockholm, Sweden; Department of Clinical Neuroscience, Karolinska Institutet, Stockholm, Sweden/Centre for Molecular Medicine, Karolinska University Hospital, Stockholm, Sweden

**Keywords:** Cognition, natalizumab, comparative effectiveness

## Abstract

**Background:**

Cognitive impairment occurs in 40%–70% of persons with multiple sclerosis (MS).

**Objective:**

To examine the effectiveness of natalizumab compared with other disease-modifying treatments (DMTs) on improving cognition as measured by the Symbol Digit Modalities Test (SDMT).

**Methods:**

Data were collected as part of Swedish nationwide phase IV surveillance studies (2007–2020). An increase in SDMT score by ⩾10% of the difference between maximum score possible (110) and the baseline value was defined as cognitive improvement. The likelihood of improvement was compared between natalizumab-treated individuals and individuals treated with other DMTs using mixed effect logistic regression. Trend in odds of improvement was investigated using slope analyses.

**Results:**

We included 2100 persons with relapsing-remitting MS treated with natalizumab and 2622 persons treated with other DMTs. At 6 months, 45% reached improvement. The natalizumab group showed largest odds of improvement during follow-up (odds ratio: 2.3, 95% confidence interval (CI): 1.5–3.5). The odds of improvement increased by 7% (95% CI: 6–7) per month of natalizumab treatment. The equivalent estimate was 4% (95% CI: 2–5) for other monoclonal antibodies and nonsignificant for oral or platform therapies.

**Conclusion:**

Treatment with natalizumab or other monoclonal antibodies is associated with a significantly faster likelihood of cognitive improvement than platform or oral DMTs.

## Introduction

Cognitive decline occurs in 40–70% of persons with multiple sclerosis (MS).^1^ It is associated with stress, decline in standards of living, loss of employment, and withdrawal from social activities.^2,3^ Cognitive impairment occurs in all stages of the disease^4,5^ often independent of physical disability.^6^

Natalizumab is a humanized monoclonal antibody^7^ used as a highly potent disease-modifying treatment (DMT) in persons with active MS. Natalizumab has been shown to efficiently reduce the risk of sustained progression of physical disability and the rate of clinical relapses in relapsing-remitting multiple sclerosis (RRMS).^8^

Studies have also shown that natalizumab decreases the risk of cognitive decline and is associated with clinically meaningful improvement in cognition in individuals with MS.^9–12^ However, the findings are mainly reported in non-randomized observational studies with short-term follow-ups and small sample sizes. In general, evidence on the effect of DMTs on cognitive improvement in MS is lacking, particularly from randomized controlled trials.^13^ Furthermore, no study has investigated the comparative effectiveness of natalizumab on cognition with other DMTs. Hence, the objective of this study was to determine and compare the long-term effectiveness of natalizumab on cognition as measured by the Symbol Digit Modalities Test (SDMT)^14,15^ in a large population-based cohort of individuals with MS. The SDMT has been shown to be associated with several clinical and patient-centered outcomes in MS, such as income, employment, and daily activity.^3,16,17^ In MS, performance on the SDMT is shown to be predictive of future cognitive decline.^18^ The SDMT has been found to be the most sensitive individual cognitive measure for use in MS.^19,20^ In this work, we examined the effectiveness of natalizumab on the improvement of cognition and compared its effect to the effects seen by using platform therapies, other monoclonal antibodies, and oral DMTs.

## Method

### Data source

Data for this study were collected from a Swedish post-market surveillance study of the long-term effectiveness and safety of DMTs, which started in 2006, the *Immunomodulation and MS Epidemiology* (*IMSE*) study.^21,22^ The IMSE cohorts are prospective recruitments of all individuals with MS throughout Sweden who start on natalizumab, fingolimod, alemtuzumab, teriflunomid, dimetyl fumarate, peginterferon beta-1a, rituximab, daclizumab, ocrelizumab, or cladribin. Based on total sold doses, it has been estimated that more than 95% of the Swedish MS patients who started on natalizumab are part of the IMSE cohort.^21^ In IMSE, patients are evaluated at baseline (treatment initiation), 6 months and annually thereafter until treatment discontinuation. A neurologist performs clinical evaluation at each time point, which includes recording of number of relapses, Expanded Disability Status Scale (EDSS) score, and possible adverse events. A trained MS nurse performs the SDMT as well as performing the blood sampling. Data collection, storage, and reports of adverse events are performed using the infrastructure provided by the Swedish MS Registry (SMSreg) web platform.^23^

### Data availability

Data related to this article are available from Tomas Olsson, Karolinska Institutet. To share data from the IMSE cohorts and Swedish MS registry, a data transfer agreement must be completed between Karolinska Institutet and the institution requesting data access. This is in accordance with the data protection legislation in Europe (General Data Protection Regulation [GDPR]). Persons interested in obtaining access to the data should contact Ali Manouchehrinia (ali.manouchehrinia@ki.se).

### Study population

Included in this study were RRMS participants from the IMSE natalizumab-treated cohort, treated with natalizumab for minimum of 6 months and naïve to treatment with other monoclonal antibodies and a randomly selected population of natalizumab-naïve individuals with MS who had received other therapies (natalizumab naïve) identified from the Swedish MS registry for the comparative effectiveness analysis. Treatment categories were defined in accordance with categorization used previously^24^ as other monoclonal antibodies (rituximab (or biosimilars), ocrelizumab, alemtuzumab, daclizumab, and ofatumumab), oral DMTs (teriflunomide, fingolimod, cladribine, dimethyl fumarate, siponimod, and ozanimod), and platform therapies (interferon beta-1a, interferon beta-1b, peginterferon beta-1a, glatiramer acetate, and peginterferon). All included individuals were required to have (1) no SDMT test performed prior to the baseline SDMT test to avoid learning effects, (2) baseline SDMT test performed at most 90 days prior to respective treatment initiation, (3) baseline SDMT score ⩾24 and ⩽90 (mean ± 2 standard deviations) to reduce potential reporting errors caused by outliers, and (4) at least three SDMT tests performed during treatment. All included participants had MS fulfilling the McDonald criteria^23–25^ as judged by their neurologist.

### Study outcome

We evaluated cognition by investigating the changes in SDMT performance over time, specifically likelihood of cognitive improvement at each follow-up visit. The SDMT ranges from 0 to 110, with a higher score indicating better cognitive performance. SDMTs are completed at baseline, at 6th month, and on an approximately annual basis during treatment with natalizumab or other therapies (natalizumab naïve). Because MS centers performed the SDMT test as either an oral or a written test, giving rise to some inconsistency in the score, we included follow-up periods of only one SDMT type (either oral or written tests) and all statistical analyses were controlled for the type of test that each participant completed. Cognitive improvement was defined as an increase in the SDMT score by ⩾10% of the difference between the maximum possible score (110) and the baseline SDMT score (at the time of treatment initiation). That is ⩾6 SDMT score improvement for a person with a baseline score of 50 and ⩾5 SDMT score improvement for a person with a baseline score of 60. The choice of relative cut-off for measuring improvement (as opposed to a fixed ⩾4- or ⩾8-point improvement) prevented ceiling effect, obscuring the improvement in persons with relatively high SDMT scores at baseline.

### Statistical analysis

We assessed the impact of natalizumab and other therapies on the likelihood of cognitive improvement using two methods. First, we started by investigating the proportion of patients treated with natalizumab who reached cognitive improvement (see above) at 6 months up to 126 months post-treatment initiation. We compared the basic clinical and demographic characteristics of patients who reached cognitive improvement at 6 months post-treatment initiation compared to those who did not show improvement using parametric or non-parametric tests.

We then proceeded to evaluate the effect of natalizumab on likelihood of cognitive improvement by fitting (1) a mixed-effects logistic regression model to obtain the likelihood (in terms of odds ratios) of cognitive improvement over the follow-up time and (2) simple slope analyses with treatment and time interaction to calculate the odds of cognitive improvement per each follow-up month for natalizumab-treated persons and persons treated with other treatments. All models were adjusted for sex, age at onset of MS, age at treatment initiation, baseline SDMT score, presence of relapse within 120 days of test, number of tests performed during follow-up, time for follow-up, and type of SDMT test performed.

We finally investigated the differences in the proportion of persons with cognitive improvement at each follow-up time between treatment groups using the chi-square tests.

To ensure the robustness of the findings, we conducted three sensitivity analyses. In the first analysis, similar models but on a propensity score-matched population was fitted. The second sensitivity analysis was performed on a subset of individuals from the original study population who were classified as cognitively impaired at baseline (the baseline SDMT score <1.5 standard deviations of sample mean). The third sensitivity analysis was performed on those who performed only oral version of the SDMT test.

All statistical analyses were performed using R version 4.0. Ethical approval was obtained from the Stockholm ethical committee (EPN) at Karolinska Institutet.

## Results

A summary of the demographic and clinical characteristics of the study population is presented in Table 1. In total, 3538 persons with MS had ever been treated with natalizumab in Sweden between 2007 and 2020 and were part of the IMSE cohort. We had to exclude 1438 patients as they did not meet the criteria to be included. From the 1438 excluded patients, 83 were excluded as they had been exposed to other monoclonal antibodies before natalizumab initiation, 277 had no SDMT performed during treatment period, 342 had previously performed SDMT before natalizumab initiation, 374 had performed <3 SDMT during follow-up, for 10 the type of SDMT test could not be determined, 225 were not RRMS, 72 had missing onset date, and 55 had their baseline SDMT score <24 or >90. This left us with a final study population comprised of 2100 patients recruited from 47 MS specialist clinics throughout Sweden. These patients had performed 17,387 SDMT tests over an average follow-up time of 54 (range: 6–156) months.

**Table 1.** Clinical and demographic characteristics of the natalizumab-treated individuals.

	Overall (*N* = 2100)
Sex	
Female	1504 (71.6%)
Male	596 (28.4%)
Age at MS onset	
Mean (SD)	28.30 (8.79)
First SDMT score	
Median (Q1, Q3)	51 (44, 58)
Age at baseline	
Mean (SD)	34.97 (9.73)
Duration of treatment (follow-up), months	
Median (Q1, Q3)	54 (30, 78)
Number of SDMT score performed during treatment	
Median (Q1, Q3)	7 (5, 9)
Type of SDMT test performed	
Oral only	1613 (76.8%)
Written only	487 (23.2%)
Duration of exposure to platform DMTs before study entry (months)*	
Median (Q1, Q3)	15.5 (0, 48)
EDSS score at baseline	
N-Miss	594
Median (Q1, Q3)	2.50 (1.5, 3.5)

SDMT: Symbol Digit Modalities Test; SD: standard deviation.

* Includes interferon beta-1a, interferon beta-1b, glatiramer acetate and peginterferon.

### The effect of natalizumab on SDMT score

**Figure 1.**
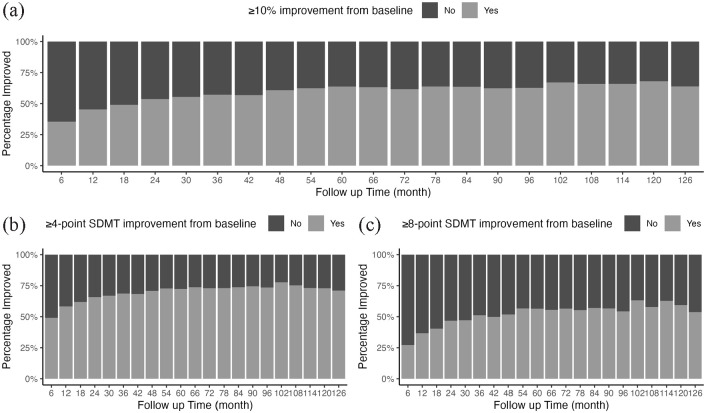
Proportion of patients with (a): 10% improvement (an increase of SDMT score by ⩾10% of the difference between the maximum possible score (110) and the baseline value), (b): ⩾4-point SDMT improvement from baseline value and (c): ⩾8-point improvement from the baseline value after treatment initiation with natalizumab.

Natalizumab treatment was associated with a significant improvement in the SDMT performance. Approximately 45% of patients (806 persons of 1779 with SDMT score at 6 month) reached improvement (increase in the SDMT score by ⩾10% of the difference between maximum possible score and the baseline score) at 6 months after treatment initiation (Figure 1(a)). Figure 1(b) and (c) illustrate percentage of natalizumab-treated patients reaching ⩾4-point and ⩾8-point SDMT improvement over the treatment with natalizumab, respectively. General demographic and baseline characteristics of those with and without cognitive improvement at 6-month post-natalizumab treatment are described in Table 2.

**Table 2.** Comparison of general baseline demographic and clinical characteristics of patients, 6 months post-natalizumab treatment stratified by cognitive improvement status.

	Did not improve (*N* = 973)	Improved (*N* = 806)	*P* value
Sex			0.310
Female	680 (69.9%)	581 (72.1%)	
Male	293 (30.1%)	225 (27.9%)	
Age at MS onset			0.002
Mean (SD)	29.20 (9.13)	27.74 (8.49)	
Age at baseline			<0.001
Mean (SD)	36.24 (9.92)	33.73 (9.55)	
Number of SDMT score performed during treatment			0.718
Median (Q1, Q3)	7 (5, 10)	7 (5, 10)	
First SDMT score			0.54
Median (Q1, Q3)	51 (44, 59)	51 (44, 58)	
Duration of treatment (follow-up), months			<0.001
Median (Q1, Q3)	48 (24, 78)	60 (30, 78)	
Duration of exposure to platform DMTs before study entry (months)*			0.053
Median (Q1, Q3)	17 (0, 51)	15 (0, 45)	
EDSS score at baseline			0.001
Number of missing	226	275	
Median (Q1, Q3)	2.5 (1.5, 3.5)	2.0 (1.5, 3.0)	

SDMT: Symbol Digit Modalities Test; SD: standard deviation.

Improvement: An increase of SDMT score by ⩾10% of the difference between the maximum score possible (110) and the baseline value.

* Includes interferon beta-1a, interferon beta-1b, glatiramer acetate and peginterferon.

### Comparative effectiveness

**Figure 2.**
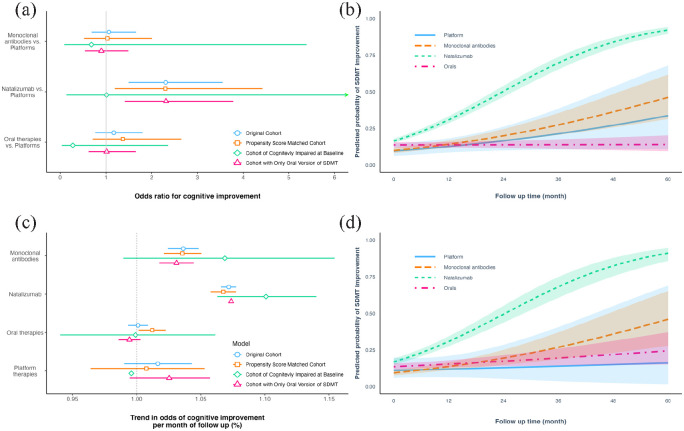
(a) Forest plot of odds ratios for cognitive improvement obtained from multivariable adjusted logistic mixed-effect models. (b) Predicted odds of reaching cognitive improvement in different treatment categories before propensity score matching. (c) Trend in odds of cognitive improvement per month of follow-up (slope analysis). Estimates indicate the percentage increase in the probability of improvement per month. (d) Predicted odds of reaching cognitive improvement after propensity score matching. Cognitive improvement was defined as an increase of SDMT score by ⩾10% of the difference between the maximum possible score (110) and the baseline value. SDMT = Symbol Digit Modalities Test.

Characteristics of the natalizumab-treated and natalizumab-naïve individuals, before and after matching, are presented in Table 3. On average, the odds of cognitive improvement was 2.3 times (95% CI: 1.5–3.5) higher in natalizumab-treated individuals compared to individuals treated with platform DMTs (the reference category). The corresponding odds ratios were 1.06 (95% CI: 0.7–1.6) for patients treated with other monoclonal antibodies and 1.2 (95% CI: 0.7–1.8) for patients on oral therapies, respectively (compared to platform DMTs) (Figure 2(a) and (b)). Younger age at MS onset and younger age at SDMT performance were positively associated with an increased probability of cognitive improvement. Inversely, the presence of a relapse within 120 days of SDMT test, and a higher baseline SDMT score decreased the probability of improvement.

**Table 3.** Clinical characteristics of the natalizumab-treated and natalizumab-naïve populations before and after matching.

	Natalizumab treated (*N* = 2100)	Never exposed to natalizumab (*N* = 2622)	Standardized mean difference	Natalizumab treated (*N* = 1077)	Never exposed to natalizumab (*N* = 1524)	Standardized mean difference
Sex						
Female	1504 (71.6%)	1797 (68.5%)	0.07	755 (70.1%)	1067 (70.0%)	0
Male	596 (28.4%)	825 (31.5%)	−0.07	322 (29.9%)	457 (30.0%)	0
Age at MS onset						
Mean (SD)	28.30 (8.79)	32.17 (9.91)	−0.44	29.68 (9.19)	30.07 (9.33)	0.03
Age at baseline						
Mean (SD)	34.97 (9.73)	40.04 (10.47)	−0.52	36.46 (10.12)	37.11 (10.05)	0.02
Duration of treatment (follow-up), months						
Median (Q1, Q3)	48 (30, 60)	30 (24, 48)	0.51	36 (18, 54)	30 (24, 48)	−0.01
Type of SDMT test performed						
Oral only	1613 (76.8%)	2148 (81.9%)	−0.12	856 (79.5%)	1234 (81.0%)	−0.03
Written only	487 (23.2%)	474 (18.1%)	0.12	221 (20.5%)	290 (19.0%)	0.03
First SDMT score						
Median (Q1, Q3)	51 (44, 58)	52 (45, 59)	−0.08	51 (44, 59)	51.5 (45, 58)	0.01
Number of SDMT score performed during treatment						
Median (Q1, Q3)	7 (5, 9)	4 (3, 5)	0.95	4 (3, 6)	7 (5, 9)	0.01
Treatment type (*n*)						
Natalizumab	2100	0		1077	0	
Monoclonal antibodies^a^	0	892		0	589	
Oral DMTs^b^	0	1541		0	865	
Platform DMTs^c^	0	189		0	70	

SDMT: Symbol Digit Modalities Test, SD: standard deviation.

a Rituximab (or biosimilars), ocrelizumab, alemtuzumab, daclizumab, and ofatumumab.

b Teriflunomide, fingolimod, cladribine, dimethyl fumarate, siponimod, and ozanimod.

c Interferon beta-1a, interferon beta-1b, glatiramer, and peginterferon beta-1a.

**Figure 3.**
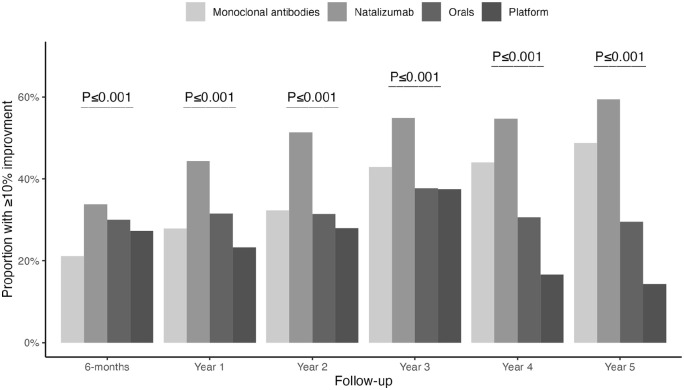
Comparison of the proportion of patients with cognitive improvement at each year of follow-up between treatment groups using the chi-square test.

The odds of cognitive improvement (slope analysis) for natalizumab-treated individuals increased by 7% (95% CI: 6–7) per month of therapy (follow-up). The equivalent estimates were 4% (95% CI: 2–5) for other monoclonal antibodies, 2% (95% CI: –1 to 4) for platform DMTs, and 1% (95% CI: –1 to 1) in those receiving oral DMTs (Figure 2(c)). Progression with time in proportion of individuals with cognitive improvement at each year of follow-up and its comparison between groups are shown in Figure 3.

One hundred and seventy-eight natalizumab-treated individuals, 58 individuals treated with other monoclonal antibodies, 64 treated with oral DMTs, and 9 with platform therapies (*n* = 309) were classified as being cognitively impaired at baseline (baseline SDMT score < 1.5 standard deviation of cohort mean). While we did not observe an overall difference between treatments in odds of improvement over the follow-up time (most likely due to small sample size) (Figure 2(a)), the odds of cognitive improvement in the natalizumab group increased by 10% (95% CI: 6–13) per month of therapy. The equivalent estimates were 7% (95% CI: –1 to 14) for other monoclonal antibodies, 0% (95% CI: 0–0) for platform DMTs and 0% (95% CI: –6 to 6) in those receiving oral therapies (Figure 2(c)). We did not see an effect of SDMT test type (oral vs written test) on the overall findings (Figure 2(a)).

### Propensity score matching analysis

In summary, similar results to those obtained before matching were found after propensity score matching (Figure 2(a) and (d)). For 1077 of the 2100 natalizumab-treated individuals, we could find 1524 propensity score-matched individuals treated with other therapies but never with natalizumab (Table 3). The two groups were matched for sex, age at MS onset, age at baseline SDMT test, baseline SDMT score, type of SDMT test (oral or written), total number of SDMTs performed, and duration of follow-up time. The odds of cognitive improvement for natalizumab-treated patients increased by 7% (95% CI: 6–7) per each month of follow-up and were 4% (95% CI: 2–5) for other monoclonal antibodies, 1% (95% CI: –6 to 5) for platform therapies and 1% (95% CI: 0–2) for oral DTMs (Figure 2(c)).

## Discussion

Using a large, nationwide population-based cohort of persons with RRMS treated with natalizumab, we found that treatment with natalizumab or other monoclonal antibodies was associated with a significantly better rate of improvement in cognition. Natalizumab and other monoclonal antibodies showed superior performance in improving cognition compared to platform therapies or oral DMTs during the follow-up time. While the trend in progression of odds of cognitive improvement was significantly higher in those receiving natalizumab and other monoclonal antibodies compared to those on platform or oral therapies, we observed an overall greater odds of having cognitive improvement in the natalizumab-treated individuals. These results remained mainly unchanged after a rigorous propensity score matching analysis. We also found beneficial effects of natalizumab and other monoclonal antibodies in improving cognition in those with major cognitive impairments, albeit with smaller effect sizes.

Despite the availability of DMTs for more than two decades, their effect on cognitive decline in MS is mostly unknown. A recent systematic review and meta-analysis of the effect of DMTs on cognitive performance in RRMS showed a minimal positive effect of DMTs on SDMT score.^25^ Even though the study included 17 (out of 44 included studies) randomized control trials, no statistically significant difference between platform and highly potent DMTs was observed. A similar conclusion has been reached in a recent systematic review of studies investigating the effects of DMT on cognition in MS.^13^ Contrary to a meta-analysis which may carry forward the limitation of included studies,^26^ the results from this study suggest that monoclonal antibodies can improve cognition in MS compared with the platform or oral therapies. Our result is consistent with results of smaller previous observational studies showing significant improvements in cognitive function after natalizumab treatment.^27–29^

The majority of patients with MS show a similar progressive decline of cognition^1^ as seen for physical disability, and both physical and cognitive deteriorations exhibit marked between and inter-patient variability. Hence, evaluating the effect of different treatments on cognition is important to improve the possibility of choosing a favorable treatment for each patient.

Former studies have used an improvement of ⩾4 SDMT score from baseline as a definition of reaching clinically meaningful improvement. We found that this approach gives individuals with a lower baseline SDMT artificially higher chance of reaching clinically meaningful improvement as it is easier to improve ⩾4 when they score lower at baseline. To handle this problem, we used the definition of cognitive improvement as an increase in SDMT by ⩾10% of the difference between maximum possible score and the baseline score which makes the impact of baseline SDMT score on cognitive improvement less significant. The learning effect, caused by repeated testing, could influence the performance on SDMT score. However, in a comparative study design, this should affect all groups equally to bias the estimates. As the models and the case–control propensity score matching were controlled for number of tests performed and duration of follow-up (i.e. treatment duration), we therefore do not think that the learning effect has significantly affected the results of our analyses.

Strengths of this study include a large nationwide population-based cohort of MS patients treated with natalizumab, longitudinal follow-up, multiple analysis methods with similar results and a large, population-based, matched control cohort of MS patients never treated with natalizumab. However, this study has some limitations. Due to cohort matching, we had to exclude many participants. We did not have information and therefore did not control for the effect of educational level, socioeconomic factors and other factors that could potentially influence cognition, which could impact the SDMT performance. However, given the Swedish population’s socioeconomic and educational homogeneity, we do not think these factors have substantially confounded our results. Furthermore, this study examined the effect of natalizumab on cognition in persons with MS as one homogeneous population and compared to patients on other DMTs. Although all patients had relapsing-remitting course and active disease, it is possible that type and severity of cognitive dysfunction may have been unevenly distributed in this cohort. It is quite likely that the characteristics of the cognitive problems affected the choice of therapy in the first place which could interfere with the results of this study beyond what our analyses could be adjusted for. Therefore, additional studies and ideally randomized trials are needed to examine further the impact of different DMTs on cognition in MS.

In conclusion, we observed that treatment with natalizumab and other monoclonal antibodies is associated with significant improvement in cognitive performance in persons with RRMS as measured by the SDMT score. The effect of these treatments on cognitive improvement was superior to the platform or oral therapies.
